# Mechanisms of Speciation

**DOI:** 10.1155/2012/820358

**Published:** 2012-05-09

**Authors:** Kyoichi Sawamura, Chau-Ti Ting, Artyom Kopp, Leonie C. Moyle

**Affiliations:** ^1^Graduate School of Life and Environmental Sciences, University of Tsukuba, Tsukuba 305-8572, Japan; ^2^Department of Life Science, National Taiwan University, Taipei 10617, Taiwan; ^3^Department of Evolution and Ecology, University of California, Davis, Davis, CA 95616, USA; ^4^Department of Biology, Indiana University, Bloomington, IN 47405, USA

“Speciation” was largely “speculation” two decades ago, at least with respect to a detailed and comprehensive mechanistic understanding of the origin of new species. Despite elegant classical work examining the genetic basis of interspecific differences and reproductive isolation and complementary studies of the ecological factors that can contribute to species divergence, speciation researchers lacked the tools to dissect the specific forces, traits, and genes involved. Thanks to the recent advances in molecular biology and genomic sequencing, detailed study of speciation is becoming feasible in many animal and plant groups. In fact, a dozen of “speciation genes” responsible for reproductive isolation between sibling species have been identified at the molecular level. Further, genetic changes leading to morphological differentiation among related species have been elucidated, supported by phylogenetic analyses at high resolution. We invited investigators to contribute both original research and review articles that would stimulate the continuing efforts to understand speciation and species differentiation from all perspectives, not only the genetic mechanisms but also the ecological and evolutionary causes.

Among the seven original articles in this special issue, four were *Drosophila* studies. Two of them focus on the mechanisms of reproductive isolation. A. Takahashi et al. in “*Cuticular hydrocarbon content that affects male mate preference of Drosophila melanogaster from west Africa*” identified a polymorphic chemical cue involved in mate recognition between sibling species. Y. H. Ahmed-Braimah and B. F. McAllister in “*Rapid evolution of assortative fertilization between recently allopatric species of Drosophila*” described an example of postmating/prezygotic isolation, where heterospecific fertilization after mating is compromised due to disruptions in sperm storage and motility. Often, reproductive isolation evolves more rapidly than any morphological traits so that the only way to distinguish recently diverged species is through mating experiments. Y.-F. Li et al. in “*DNA barcoding and molecular phylogeny of Drosophila lini and its sibling species*” showed that molecular variation can also be widely shared between sibling species despite strong reproductive isolation between them. To understand why reproductive isolation can evolve so rapidly compared to other traits, L. Müller et al. in “*Inter- and intraspecific variation in Drosophila genes with sex-biased expression*” examined the evolution of gene expression and protein sequences and found that genes with male-biased expression tend to diverge rapidly compared to the rest of the genome.

Three other original articles deal with reproductive isolation in vertebrates, specifically fish or reptiles. D. Bierbach et al. in “*Divergent evolution of male aggressive behavior*:* another reproductive isolation barrier in extremophile poeciliid fishes*?” reported a rare case study of behavioral isolation via local adaptation to extreme environmental conditions (darkness in caves and toxic hydrogen sulphide). G. M. Kozak et al. in “*Postzygotic isolation evolves before prezygotic isolation between fresh and saltwater populations of the rainwater killifish, Lucania parva”* discovered a case of incipient reproductive isolation caused by salinity adaption, in which they found no evidence of prezygotic isolation but detected reduced hybrid survival. M. Gabirot et al. in “*Differences in chemical sexual signals may promote reproductive isolation and cryptic speciation between Iberian wall lizard populations*” reported another case of cryptic speciation caused by pheromonal differentiation, in which they demonstrated that the Iberian wall lizard forms part of a “species complex” with different morphology and different proportions of chemical components in femoral gland secretions of males.

This special issue also includes four review articles. A. Ivanović et al. in “*A phenotypic point of view of the adaptive radiation of crested newts *(*Triturus cristatus superspecies, Caudata, Amphibia*)” reviewed the pattern of adaptive radiation in the European crested newt, in which they suggested that phenotypic diversification was caused by heterochronic changes linked to variation in ecological preferences. J. P. Masly in “*170 years of “lock-and-key”*:* genital morphology and reproductive isolation*” reviewed the facts and speculations about the role of genital morphology in maintaining species barriers and examined the prospects for identifying the genetic changes responsible for the evolution of genital morphology. D. M. Castillo and L. C. Moyle in “*Evolutionary implications of mechanistic models of TE-mediated hybrid incompatibility*” reviewed mechanistic models of host-mediated TE suppression in light of the potential role of TE derepression in postzygotic isolation and identified data that would be necessary to provide more satisfactory tests of this hypothesized isolation mechanism. K. Sawamura in “*Chromatin evolution and molecular drive in speciation*” proposed a general mechanism of hybrid sterility and inviability caused by coevolution between repetitive satellite DNAs and chromatin proteins.

Altogether, this is a diverse array of papers that ranges from a classical model of speciation research—*Drosophila*—to plants and emerging vertebrate system, and from molecular genetic mechanisms through sensory biology to environmentally mediated adaptive divergence. As such, this special issue is representative of the diversity of studies and systems that continue to contribute to our understanding of speciation and the diversity of mechanisms that surely underlie this fundamental evolutionary process. We can only hope that the field, and our understanding, continues to grow so diversely and creatively in the future. 

